# Decellularized testicular bio-scaffolds drive *in vitro* differentiation of chemically reprogrammed fibroblasts

**DOI:** 10.3389/fbioe.2026.1900213

**Published:** 2026-08-18

**Authors:** Sharon Arcuri, Georgia Pennarossa, Tiziana A. L. Brevini, Fulvio Gandolfi

**Affiliations:** 1 Department of Veterinary Medicine and Animal Science, Laboratory of Biomedical Embryology and Tissue Engineering, Center for Stem Cell Research, Università degli Studi di Milano, Milan, Italy; 2 Department of Agricultural and Environmental Sciences - Production, Landscape, Agroenergy, Università degli Studi di Milano, Milan, Italy

**Keywords:** 3D scaffolds, chemical reprogramming, decellularization, male infertility, testis

## Abstract

Spermatogenesis is a highly orchestrated and complex biological process that, to date, has only been partially reproduced *in vitro*, primarily in murine models. Despite the growing prevalence of human male infertility, its etiology remains poorly understood, largely due to the limited availability of physiologically relevant experimental models and the intrinsic inaccessibility of human testicular tissue. The development of advanced 3D *in vitro* systems capable of reproducing the structural, biomechanical, and biochemical features of the testicular microenvironment represents a critical step toward modeling human spermatogenesis and dissecting the molecular mechanisms underlying male infertility. Here, we describe a novel bioengineering strategy that integrates chemical reprogramming cues with tissue-specific mechanical signals to generate biomimetic 3D testicular models. To this purpose, testicular bio-scaffolds were produced from bovine testes using a three-step decellularization protocol consisting of a freeze–thaw cycle followed by sequential treatments with 0.3% sodium dodecyl sulfate (SDS) for 12 h and 1% Triton X-100 for 6 h. The generated decellularized testicular bio-scaffolds preserved the gross morphology of the native tissue. Histological and DAPI staining, together with DNA quantification analyses, confirmed the effective removal of cellular material, with low residual DNA levels, compared with untreated testis. Histochemical analyses demonstrated the preservation of the key extracellular matrix (ECM) components collagen, elastin, and glycosaminoglycans, ensuring retention of the tissue-specific microarchitecture. SEM confirmed preservation of the 3D ultrastructure of the extracellular matrix, while quantification of residual SDS demonstrated efficient detergent removal. The resulting acellular scaffolds were subsequently repopulated with 1 x 10^6^ chemically reprogrammed human skin fibroblasts/mm^3^. Seven days after engraftment, cells had turned down the expression of pluripotency markers and actively transcribed for the testicular-associated markers SOX9, SS18L1, AMH, UCHL1, and PRM1. Immunofluorescence analyses further confirmed the expression of SOX9, AMH, and UCHL1 proteins, supporting the acquisition of testicular-associated phenotypes within the repopulated bio-scaffolds. Overall, these findings demonstrate that the generated decellularized testicular bio-scaffold represents a promising biomimetic artificial niche able to recapitulate key aspects of the native microenvironment. This platform may provide a reliable and versatile tool for modeling human spermatogenesis, investigating the pathophysiology of male infertility, and supporting future regenerative and translational applications.

## 1 Introduction

Spermatogenesis is a highly coordinated multi-stage process that takes place within the specialized microenvironment of the testis, where germ cells interact dynamically with somatic cells and the extracellular matrix (ECM) to generate haploid male gametes. *In vivo*, this process is regulated by a complex interplay of endocrine, paracrine, and autocrine signals together with finely tuned biomechanical cues operating within the seminiferous tubules (Oatley and Brinster, 2012; Griswold, 2016). The spatial organization of the seminiferous epithelium, ECM composition and stiffness, together with signaling gradients, maintain germ cell homeostasis and support lineage commitment, meiosis, and terminal differentiation. Disruption of this tightly regulated system can impair germ-cell development and may lead to male infertility, a condition affecting approximately 7% of men worldwide and representing an increasing global health concern (Tahmasbpour et al., 2014; Agarwal et al., 2021). Despite its high prevalence, the mechanisms underlying impaired spermatogenesis remain poorly understood. This is largely due to the intricate architecture of the testis and the highly dynamic interactions among its cellular and molecular components that are difficult to reproduce experimentally. Indeed, currently available *in vitro* and animal models still present important limitations (Huleihel et al., 2007; Oliver and Stukenborg, 2020; Bashiri et al., 2023; Salem et al., 2023; Gizer et al., 2024). Although animal studies have provided fundamental insights into testicular physiology, interspecies differences often limit the translation of findings to human reproductive biology. Likewise, conventional two-dimensional (2D) culture systems fail to reproduce the structural and biochemical complexity of the native testicular niche, including the three-dimensional (3D) ECM organization and the extensive cell-to-cell interactions required for proper tissue function (Reda et al., 2014; Neto et al., 2016; Sakib et al., 2020). As a result, the development of physiologically relevant platforms capable of mimicking the human testicular microenvironment has become a major objective in reproductive research.

In this context, recent advances in tissue engineering and 3D culture technologies have introduced several approaches, including organoids (Richer et al., 2020), hydrogel-based supports (Stukenborg et al., 2008; Perrard et al., 2016), and microfluidic platforms (Alves-Lopes and Stukenborg, 2018). Nevertheless, these models still only partially reproduce the native testicular environment, particularly the complex interplay among somatic cells, germ cells, and ECM components, along with the dynamic biochemical gradients present *in vivo* (Baert et al., 2017). Among the several bioengineering approaches, decellularized tissue-derived bio-scaffolds have emerged as particularly appealing tools because they maintain the native tissue architecture and biochemical cues while removing the immunogenic cellular material (Gilbert et al., 2006; Badylak, 2007; Crapo et al., 2011). In particular, the preservation of native ECM proteins, growth factors, and mechanical properties appears to promote more physiologically relevant cell-to-cell and cell-to-matrix interactions compared with synthetic or simplified 3D systems. These findings highlight the potential of decellularized matrices as advanced platforms for modeling tissue-specific cellular behavior and microenvironmental dynamics. However, despite these encouraging observations, the use of decellularized testicular scaffolds to recreate human spermatogenesis *in vitro* and direct testicular cell differentiation remains largely underexplored.

Here, we report the development of a novel bioengineering strategy that integrates chemically induced high plasticity with tissue-specific biomechanical and structural cues provided by the decellularized testicular ECM to generate human biomimetic 3D testicular models. To this end, bio-scaffolds were generated from bovine testes through optimized decellularization protocols (Di Filippo et al., 2025) aimed at preserving the structural integrity and biochemical composition of the native tissue. They were subsequently repopulated with human skin fibroblasts that were previously subjected to chemical reprogramming through treatment with the well-established DNA demethylating agent 5-azacytidine (5-aza-CR) (Pennarossa et al., 2013; 2021a; Brevini et al., 2014; Mirakhori et al., 2015a; 2015b; Chandrakanthan et al., 2016; Manzoni et al., 2016; Diomede et al., 2018). Maintenance of tissue architecture and ECM components in the generated bio-scaffolds was thoroughly assessed using histological and histochemical analyses. In addition, the ability of the bio-scaffolds to support the homing, attachment, and proliferation of chemically induced high-plasticity cells was evaluated through histological examinations, DNA quantification assays, and 4′,6-diamidino-2-phenylindole (DAPI) immunostaining. Finally, the capacity of the bio-scaffolds to promote the *in vitro* differentiation of chemically reprogrammed cells toward a testicular-like phenotype was investigated by analyzing the expression of key markers associated with testicular and germ cell development.

## 2 Materials and methods

All reagents were purchased from Sigma-Aldrich (Milan, Italy), unless otherwise indicated.

### 2.1 Ethical statement

Bovine testes were obtained from a local abattoir from two-year-old bulls destined for human consumption. As the tissues were collected from animals already entering the food chain, this study does not constitute animal experimentation under Directive 2010/63/EU of the European Parliament.

Human skin biopsies were obtained from healthy donors undergoing surgical procedures, following written informed consent and approval by the Ethics Committee of Ospedale Maggiore Policlinico, Milan, Italy. All procedures were conducted in accordance with the approved ethical guidelines.

### 2.2 Creation of testicular bio-scaffolds

Four bovine testes (n = 4) were collected at a local slaughterhouse and transported to the laboratory within 1 h in sterile 0.9% NaCl solution. Upon arrival, tissues were rinsed in phosphate-buffered saline (PBS) and processed according to the decellularization protocol described by Di Filippo et al. (2025). Briefly, the tunica albuginea was removed, and the testicular parenchyma was sectioned into fragments of approximately 0.5 × 0.5 × 0.5 cm^3^. Samples were then frozen at −80 °C for at least 24 h, thawed at 37 °C in a water bath for 30 min, and incubated in 0.3% (v/v) sodium dodecyl sulfate (SDS; Bio-Rad) in deionized water for 12 h, followed by incubation in 1% (v/v) Triton X-100 in deionized water for 6 h. Subsequently, samples were thoroughly washed in deionized water for 6 h, with solution changes every 2 h. At the end of the procedure, the resulting testicular bio-scaffolds were sterilized by immersion in 70% ethanol supplemented with 2% antibiotic/antimycotic solution (Thermo Fisher Scientific, Milan, Italy) in sterile water for 30 min. All procedures were performed on an orbital shaker set at 200 rpm at room temperature. Untreated native testicular tissue was used as control (CTR; n = 4).

### 2.3 Isolation and culture of human skin fibroblasts

Skin biopsies were collected from four donors (n = 4), transported to the laboratory in sterile PBS containing 2% antibiotic/antimycotic solution, and processed for fibroblast isolation. Tissues were minced into fragments of approximately 2 × 2 × 2 mm^3^ and placed onto 0.1% gelatin-pre-coated 35-mm culture dishes (Sarstedt, Milan, Italy). A drop of Dulbecco’s Modified Eagle Medium (DMEM, Thermo Fisher Scientific, Milan, Italy) supplemented with 20% fetal bovine serum (FBS, Thermo Fisher Scientific, Milan, Italy), 2 mM L-glutamine, and 2% antibiotic/antimycotic solution was added to each fragment. Cultures were performed in a humidified chamber at 37 °C in 5% CO_2_. After 6 days, fibroblasts migrated out from the skin explants, which were then removed. Isolated cells were expanded using DMEM (Thermo Fisher Scientific, Milan, Italy) supplemented with 10% FBS (Thermo Fisher Scientific, Milan, Italy), 2 mM L-glutamine, and 1% antibiotic/antimycotic solution and passaged twice per week at a 1:3 split ratio. All experiments were performed using four independent biological samples (n = 4), with each analysis carried out in triplicate in a minimum of three independent experiments.

### 2.4 Chemical reprogramming of skin fibroblasts

Primary human skin fibroblasts (n = 4) were chemically reprogrammed, as previously described (Pennarossa et al., 2013; 2020b; 2021a; 2023a; Brevini et al., 2014; 2016b; Manzoni et al., 2016). Briefly, cells were seeded in T75 culture flasks pre-coated with 0.1% gelatin 35-mm culture dishes at a density of 7.8 × 10^4^ cells/cm^2^ and cultured in DMEM (Thermo Fisher Scientific, Milan, Italy) supplemented with 10% FBS (Thermo Fisher Scientific, Milan, Italy), 2 mM L-glutamine, and 2% antibiotic/antimycotic solution under standard conditions (37 °C, 5% CO_2_, and humidified atmosphere). Twenty-four hours after plating, fibroblasts were treated with the epigenetic modifier 5-aza-CR at a final concentration of 1 μM for 18 h. At the end of incubation, chemically reprogrammed cells were used for scaffold repopulation in triplicate across at least three independent experiments. Experiments were performed using fibroblasts isolated from four independent donors (n = 4).

### 2.5 Repopulation of testicular bio-scaffolds with chemically reprogrammed fibroblasts

Testicular bio-scaffolds were placed in 96-well culture plates and pre-conditioned with DMEM (Thermo Fisher Scientific, Milan, Italy) supplemented with 10% FBS (Thermo Fisher Scientific, Milan, Italy), 2 mM L-glutamine, and 1% antibiotic/antimycotic solution for 1 h. Chemically reprogrammed human fibroblasts were then counted and seeded onto the decellularized testis at a density of 1 × 10^6^ cells/mm^3^ and allowed to attach for 2 h in a humidified incubator at 37 °C with 5% CO_2_. Additional culture medium was subsequently added to cover the bio-scaffolds, and media were replaced every 2–3 days. Repopulated scaffolds were analyzed after 1, 2, 5, and 7 days of culture.

### 2.6 DNA quantification

Total DNA content was quantified in native and decellularized testicular tissues to assess the efficiency of decellularization. Tissue samples were lyophilized and weighed, and DNA was extracted using a commercial DNA isolation kit (PureLink® Genomic DNA Kit, Thermo Fisher Scientific, Milan, Italy) following the manufacturer’s instructions. DNA concentration was measured using a NanoDrop 8000 and expressed as ng DNA per mg of tissue.

### 2.7 Cell density measurements

Decellularization efficiency and cell density within repopulated testicular bio-scaffolds was quantified using DAPI-stained sections. Fluorescent images were acquired at 200× magnification using a fluorescence microscope (Eclipse E600, Nikon, Japan) equipped with a digital camera and using NIS-Elements software (version 4.6; Nikon, Amsterdam, Netherlands). For each scaffold, at least five randomly selected fields were analyzed. Nuclei were counted using ImageJ software (version 1.53j; https://imagej.nih.gov/ij/index.html, NIH, United States), and cell density was calculated as the number of cells per unit area (cells/mm^2^). Decellularization efficiency was assessed as the reduction in nuclear density compared to that in native tissue. All measurements were performed in triplicate for each scaffold, and values were averaged across three independent experiments to ensure reproducibility.

### 2.8 Histological evaluations

Control and decellularized testicular tissues, along with the repopulated scaffolds, were fixed in 10% buffered formalin (Bio-Optica, Milan, Italy) for 24 h at room temperature. Samples were dehydrated through a graded ethanol series, cleared in xylene, and embedded in paraffin. Sections of 5-μm thickness were cut using a rotary microtome and mounted onto glass slides. Morphological assessment was performed using hematoxylin and eosin (H&E; Bio-Optica, Milan, Italy). Specific ECM components were evaluated with the following histochemical stains: Crossmon trichrome for collagen, orcein for elastic fibers, and Alcian blue/PAS for glycosaminoglycans (GAGs, Bio-Optica, Milan, Italy). Slides were examined under a light microscope (Eclipse E600, Nikon, Japan), and representative images were captured with a digital camera using NIS-Elements software (version 4.6; Nikon, Amsterdam, Netherlands). All histological evaluations were performed in triplicate for each sample.

### 2.9 Stereological analysis

Quantitative assessment of tissue architecture and ECM components in control and decellularized scaffolds was performed on paraffin-embedded sections (5 μm) stained with Crossmon trichrome, orcein, and Alcian blue/PAS. Stereological measurements were carried out using the point-counting method with a grid overlay according to established protocols (Albl et al., 2016). For each sample, at least five randomly selected fields were analyzed at 200× magnification. Volume fractions of collagen, elastic fibers, and GAGs were calculated as the ratio of points hitting the component of interest to the total number of points over the reference tissue. All analyses were performed in triplicate per sample. Data are expressed as the mean ± standard deviation (SD).

### 2.10 Assessment of residual SDS content

Residual SDS content was quantified to evaluate the efficiency of detergent removal following decellularization, as previously described by Zvarova et al. (2016). Briefly, lyophilized testicular bio-scaffolds were weighed and incubated in ultrapure water (1 mL per 10 mg dry scaffold) at 37 °C for 24 h under gentle agitation to extract residual detergent. Extracts were subsequently collected and clarified by centrifugation at 10,000 × g for 10 min. Concentration of residual SDS was determined using a methylene blue active substances (MBAS) assay. Briefly, 500 μL of scaffold extract was mixed with 250 μL of 0.0125% methylene blue solution and 500 μL of chloroform. Samples were vortexed for 30 s and centrifuged to achieve phase separation. The chloroform phase containing the methylene blue–SDS complex was collected, and absorbance was measured at 650 nm using a microplate reader. A standard calibration curve was generated using SDS solutions of known concentrations (0 μg/mL–20 μg/mL). Residual SDS levels were calculated from the calibration curve and normalized to the scaffold dry weight. Results were expressed as μg SDS/mg dry scaffold. All measurements were performed in triplicates, and data are presented as the mean ± SD.

### 2.11 Scanning electron microscopy (SEM)

Decellularized testicular bio-scaffolds were extensively rinsed in deionized water to remove residual detergents and sectioned to expose the internal architecture of the scaffold. Samples were fixed overnight at 4 °C in an aqueous solution containing 2.5% glutaraldehyde and 4% paraformaldehyde. Following fixation, tissues were dehydrated through a graded ethanol series (30%, 70%, 80%, 90%, and 100%; 15 min per step). Complete drying was achieved by sequential incubation in hexamethyldisilazane (HMDS; Merck) and ethanol mixtures (1:3, 1:1, and 3:1; 20 min each), followed by immersion in 100% HMDS overnight under a fume hood. Dried specimens were mounted on aluminum stubs using conductive carbon tape and sputter-coated with a thin gold layer (SEMPrep 2, Nanotech). The ultrastructure of the generated bio-scaffolds was examined using an FE-SEM SIGMA scanning electron microscope (Zeiss) operated at an accelerating voltage of 7 kV.

### 2.12 Cytotoxicity assessment

3(4,5-dimethylthiazole-2-yl)-2,5-diphenyltetrazolium-bromide (MTT, Roche) assay was performed on for bio-scaffolds to determine the cytotoxicity of decellularized testes. Briefly, murine fibroblasts were seeded onto flat-bottom 96-well plates at concentration of 5 × 10^3^ cells/mL (100 µL per well). After 24 h, testicular bio-scaffolds were sterilized using 70% ethanol and 2% antibiotic solution in sterile H_2_O for 30 min and extensively washed in PBS. An amount of 20 mg of decellularized testes was added to cells in triplicates and co-cultured for 1, 3, and 7 days. A measure of 10 μL of MTT solution was then added to media and incubated for 4 h. Formazan salt crystals were dissolved in 100 µL of 10% SDS in 0.01 M HCl overnight. The optical density (OD) was measured at 550 nm. The same number of cells seeded without bio-scaffolds was used as the control.

### 2.13 Gene expression analysis

Total RNA was isolated using the TaqMan™ Gene Expression Cells-to-CT™ Kit (Applied Biosystems, Milan, Italy) according to the manufacturer’s instructions. DNase I was included in the lysis buffer at a 1:100 dilution to remove genomic DNA contamination. Quantitative PCR was performed on a CFX96 Real-Time PCR system (Bio-Rad, Milan, Italy) using pre-designed gene-specific primers and probe sets from TaqMan™ Gene Expression Assays (primer details are listed in Table 1). ACTB and GAPDH were used as the internal reference genes. Target gene expression was quantified using CFX Manager software (version 18450000, Bio-Rad, Milan, Italy). Expression levels are presented relative to the highest expressed gene in the dataset, which was normalized to 1.

**TABLE 1:** List of primers used for quantitative PCR.

Gene	Description	Cat. N
ACTB	Actin, beta	Hs01060665_g1
AMH	Anti-Mullerian hormone	Hs00174915_m1
DDX4	DEAD-box helicase 4	Hs00987125_m1
DAZL	Deleted in azoospermia like	Hs00154706_m1
GAPDH	Glyceraldehyde-3-phosphate dehydrogenase	Hs02786624_g1
NANOG	Nanog homeobox	Hs02387400_g1
OCT4	POU class 5 homeobox 1	Hs04260367_gH
PRM1	Protamine 1	Hs00358158_g1
REX1	ZFP42 zinc finger protein	Hs01938187_s1
SOX2	SRY-box transcription factor 2	Hs04234836_s1
SOX9	SRY-box transcription factor 9	Hs00165814_m1
SS18L1	SS18L1 subunit of BAF chromatin remodeling complex	Hs00988004_m1
STRA8	Stimulated by retinoic acid 8	Hs00699313_g1
SYCP3	Synaptonemal complex protein 3	Hs00538146_m1
TNP1	Transition protein 1	Hs00358411_m1
THY1	Thy-1 cell surface antigen	Hs06633377_s1
UCHL1	Ubiquitin C-terminal hydrolase L1	Hs00985157_m1
VIM	Vimentin	Hs00958111_m1

### 2.14 Immunofluorescence analysis

Repopulated testicular bio-scaffolds were fixed in 10% neutral buffered formalin for 24 h, gradually dehydrated through a graded ethanol series, cleared in xylene, and embedded in paraffin. Sections (5-μm-thick) were cut using a rotary microtome, mounted onto glass slides, deparaffinized, and rehydrated. Antigen retrieval was performed using 10 mM sodium citrate buffer (pH 6.0) containing 0.05% Tween-20 at 120 °C.

Non-specific binding sites were blocked with 10% goat serum in PBS for 30 min at room temperature. Sections were then incubated with primary antibodies against SOX9 (Abcam, 1:100 dilution), AMH (Abcam, 1:100 dilution), and UCHL1 (Abcam, 1:100 dilution) for 1 h at room temperature. Following PBS washes, samples were incubated with the appropriate Alexa Fluor™ 594-conjugated and 488-conjugated secondary antibody (1:250) for 30 min. Nuclei were counterstained with 1 μg/mL DAPI.

All incubations were performed at room temperature, unless otherwise specified. At the end of the staining procedure, sections were examined using a fluorescence microscope (Eclipse E600, Nikon, Japan) equipped with a digital camera. Images were acquired using NIS-Elements software (version 4.6; Nikon, Amsterdam, Netherlands).

### 2.15 Statistical analysis

All quantitative data are presented as the mean ± SD from at least three independent experiments. Statistical analyses were performed using SPSS (version 19.1, IBM). Comparisons between two groups were conducted using unpaired Student’s t-test, while multiple group comparisons were performed using one-way ANOVA followed by Tukey’s *post hoc* test. A *p*-value <0.05 was considered statistically significant, and they are indicated by distinct superscripts.

## 3 Results

### 3.1 Generation and evaluation of testicular biological scaffolds

**FIGURE 1:**
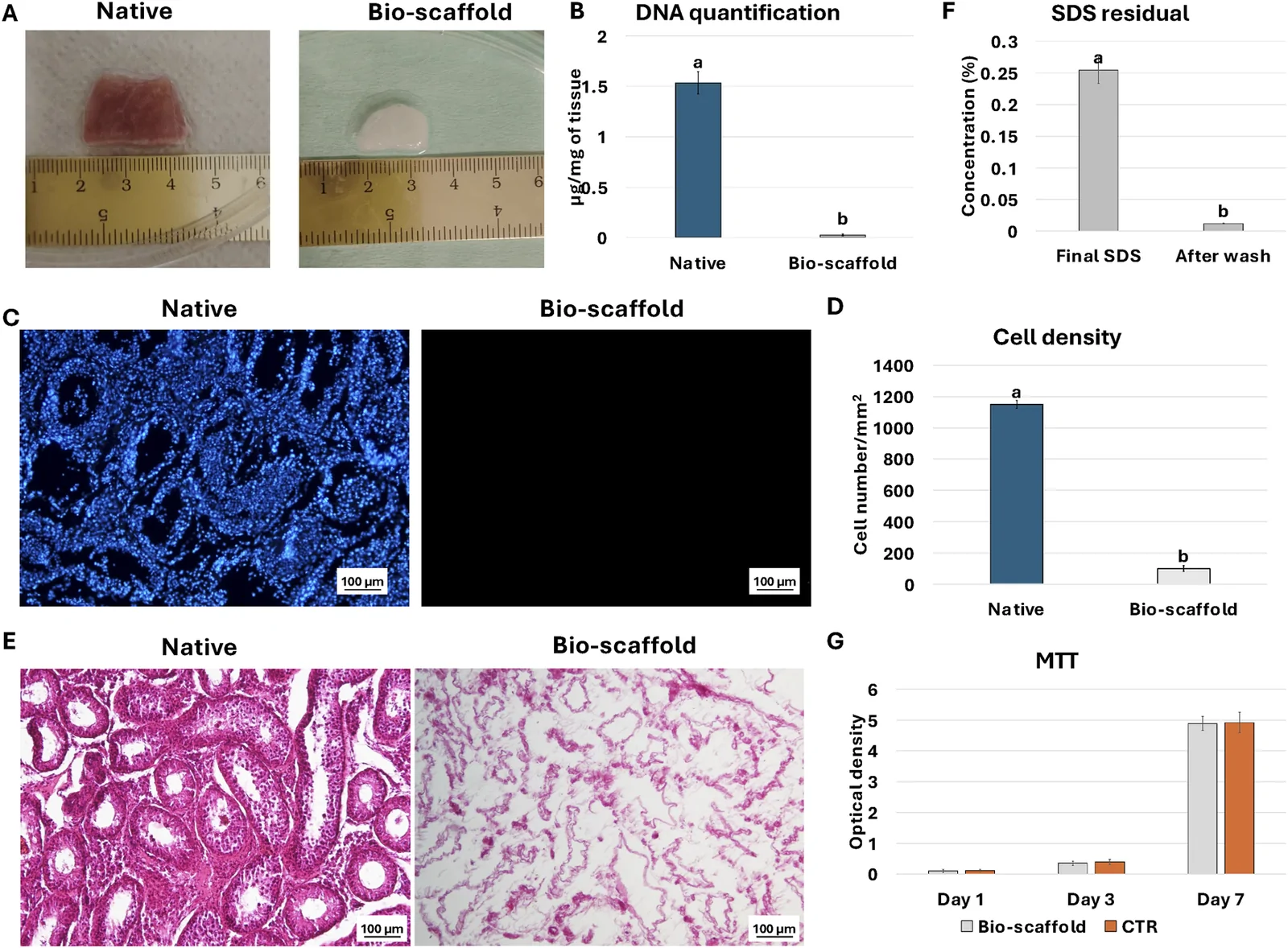
Macro/microscopic evaluations and DNA quantification in untreated (native) and decellularized (bio-scaffold) testis. **(A)** Native tissue and bio-scaffold display comparable shapes and homogeneity, while tissue color changes from red (native) to white (bio-scaffold). **(B)** DNA quantification shows a significant decrease in the bio-scaffold DNA content compared with that of the native tissue. Data are expressed as the mean ± standard deviation (SD). Different superscripts indicate a *p*-value <0.05. **(C)** DAPI staining displays the presence of nuclei in native testis and their absence after the decellularization process (bio-scaffold). Scale bars 100 μm. **(D)** Cell density demonstrates a significantly lower number of nuclei in bio-scaffold than in the untreated tissue (native). Data are expressed as the mean ± standard deviation (SD). Different superscripts indicate a *p*-value <0.05. **(E)** Hematoxylin–eosin staining shows the presence of both basophilic (cell nuclei) and eosinophilic (cell cytoplasm and ECM) staining in untreated tissue (native), while cell nuclei and the related basophilic staining are absent in the decellularized bio-scaffold. Scale bars 100 μm. **(F)** Quantification of residual SDS content demonstrates a significant reduction in detergent concentration following the washing procedure. Data are expressed as the mean ± standard deviation (SD). ^a,b^ Different superscripts indicate a *p*-value <0.05. **(G)** MTT assay demonstrates no significant differences in OD values between cells co-cultured with bio-scaffold and those of control (CTR) at the different time-points analyzed. Data are expressed as the mean ± standard deviation (SD).

Macroscopic observations revealed that at the end of the decellularization process, tissues appeared whitish and translucent compared to the native testes (Native, Figure 1A), indicating substantial loss of cellular material.

DNA quantification analysis showed a statistically significant reduction in total DNA content in the decellularized scaffolds compared to that in native controls (Native). Specifically, native testes contained 1.534 ± 0.11 μg DNA/mg, while decellularized bio-scaffolds showed a residual DNA content of 0.026 ± 0.01 μg DNA/mg, corresponding to a reduction of approximately 98.3% (p < 0.05; Figure 1B). Fluorescence analysis of DAPI-stained sections demonstrated abundant, uniformly distributed nuclei in the native testicular tissues, while decellularized scaffolds exhibited the absence of nuclear staining (Figure 1C). In addition, the quantitative measurements revealed a statistically significant reduction in nuclear density in decellularized samples compared to that in the untreated counterpart (Native, Figure 1D).

Histological evaluation by H&E staining indicated that despite the removal of cellular elements, the overall testicular architecture was preserved in the obtained scaffolds. Seminiferous tubule outlines and interstitial spaces remained clearly visible, although devoid of germinal and somatic cells. No evident structural collapse or disruption of tissue organization was observed (Figure 1E).

**FIGURE 2:**
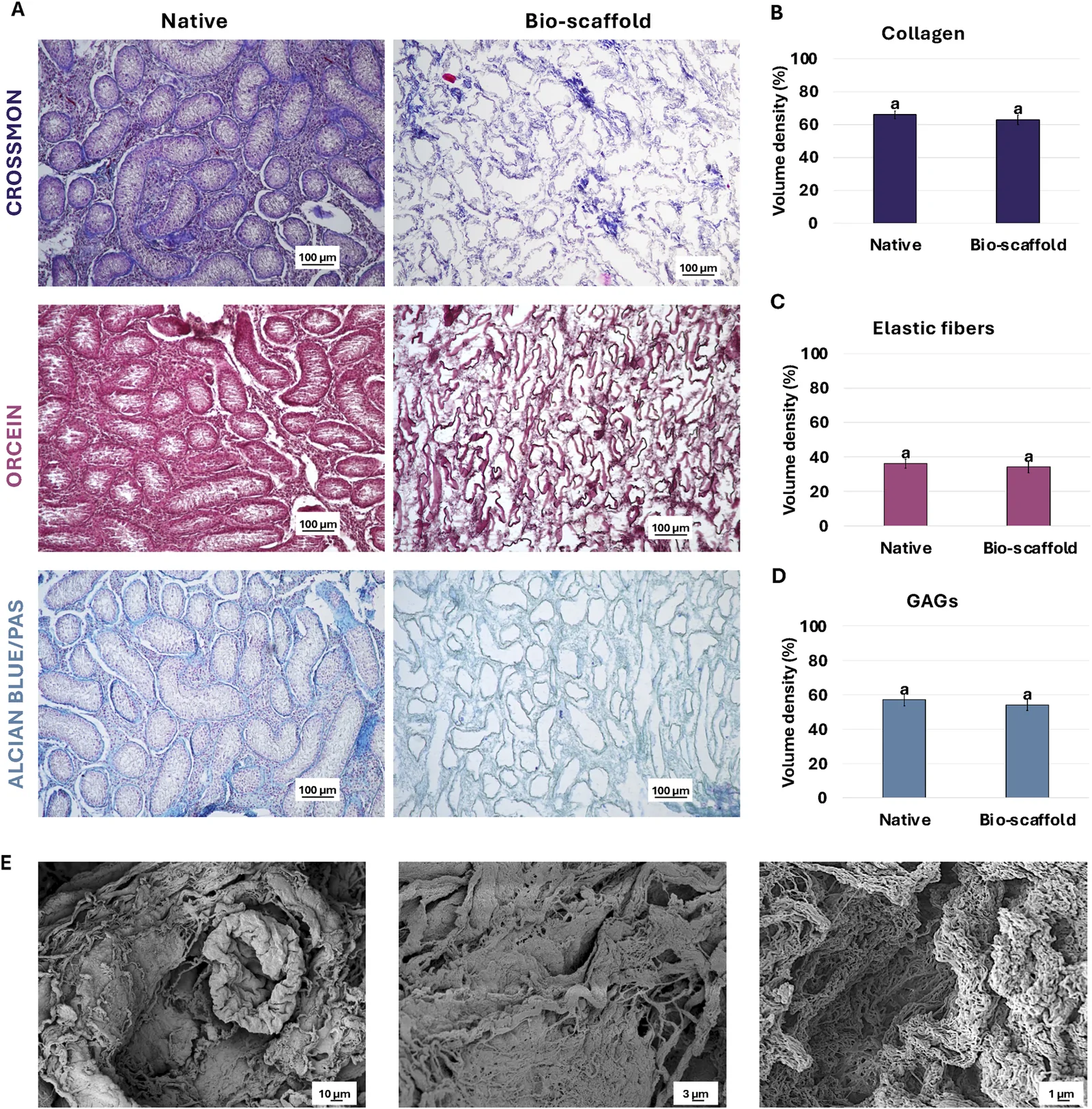
ECM microarchitecture and composition in native tissue and bio-scaffold. **(A)** Crossmon trichrome, orcein, and Alcian blue/PAS staining show the persistence and comparable distribution of collagen fibers, elastic fibers, and GAGs, respectively, in native tissue (left panels) and bio-scaffold (right panels). **(B)** Collagen content analysis demonstrates no significant differences between native and bio-scaffold groups. Data are expressed as the mean ± standard deviation (SD). **(C)** Elastin quantification shows comparable amount of the protein before (native) and after (bio-scaffold) decellularization. Data are expressed as the mean ± standard deviation (SD). **(D)** Quantitative analysis of total GAG content revealed no significant reductions in bio-scaffold compared with native testis. Data are expressed as the mean ± standard deviation (SD). **(E)** Representative SEM images showing the preservation of 3D ultrastructure and the maintenance of the seminiferous tubule architecture and the interconnected ECM network with well-organized collagen fibers. Scale bars: 10 μm, 3 μm, and 1 μm.

Histochemical staining revealed preservation of major ECM components following decellularization. In particular, Crossmon trichrome staining showed collagen fibers maintaining their distribution and organization within the interstitial and peritubular regions (Figure 2A). Orcein staining confirmed the presence of elastic fibers, which remained detectable and structurally intact after treatment (Figure 2A). Alcian blue/PAS staining demonstrated the retention of GAGs, indicating the preservation of proteoglycan-rich regions within the ECM (Figure 2A). Stereological analysis provided a quantitative assessment of ECM preservation. The volume fractions of collagen (Figure 2B), elastic fibers (Figure 2C), and GAGs (Figure 2D) in decellularized scaffolds were comparable to those observed in native tissue, with no significant differences being detected.

SEM analysis confirmed the preservation of the 3D ultrastructure of the generated testicular bio-scaffolds (Figure 2E). Low-magnification images demonstrated effective removal of the cellular compartment while preserving the overall architecture of the seminiferous tubules and interstitial regions (Figure 2E). Higher-magnification observations revealed a well-preserved ECM framework characterized by an interconnected fibrillar network and intact collagen fiber organization, maintaining a porous microenvironment that is potentially suitable for subsequent cell engraftment (Figure 2E).

Residual SDS quantification demonstrated efficient detergent removal following the decellularization procedure. In particular, a significant reduction in SDS concentration was detected after the washing steps, decreasing from 0.2546% ± 0.02121% vs. 0.0118% ± 0.00066% (Figure 1F).

MTT assay demonstrated no cytotoxic effects exerted by the generated bio-scaffold. In particular, no significant differences in OD values were detected between cells co-cultured with the decellularized testis and those of the control (CTR; Figure 1G). The two groups displayed comparable viability at day 1 and day 3 of culture (Figure 1G). In addition, even protracted exposure (7 days) indicated the absence of cytotoxic response and confirmed the efficient removal of the detergent compounds used during decellularization (Figure 1G).

### 3.2 Isolation and chemical reprogramming of human skin fibroblasts

**FIGURE 3:**
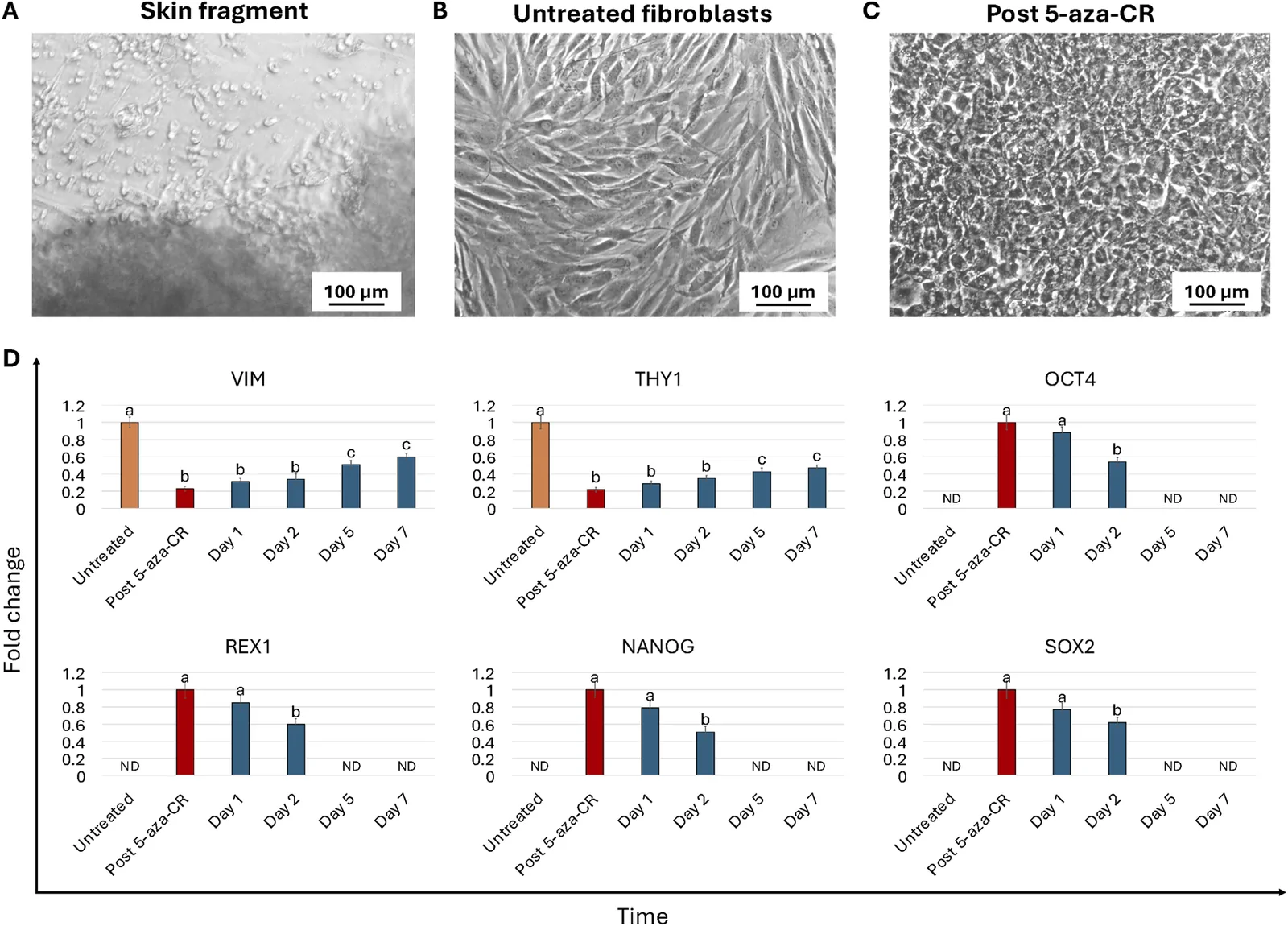
Isolation of skin fibroblasts and induction of high plasticity. **(A)** Within 6 days of culture, cells migrated out from the skin fragments. **(B)** Isolated cells exhibited a homogeneous fibroblast-like morphology. **(C)** After 5-aza-CR treatment, fibroblasts lost their typical elongated shape and became smaller in size, with granular, vacuolated cytoplasm, larger nuclei, and formed distinguishable aggregates (post-5-aza-CR, scale bar: 100 µm). **(D)** Transcription levels for the fibroblast-specific markers VIM and THY1 and the pluripotent-related genes OCT4, NANOG, REX1, and SOX2 in untreated fibroblasts (untreated), in fibroblasts exposed to 5-aza-CR (post-5-aza-CR), and at day 1, 2, 5, and 7 after seeding onto testicular bio-scaffold. Gene expression is presented with the highest expression set to 1 and all others relative to this. ^a,b,c^ Different superscripts indicate significant differences (*p* < 0.05).

Human skin fibroblasts were successfully isolated from all donor biopsies (n = 4). Within 6 days of culture, spindle-shaped cells migrated from the fragments (Figure 3A) and progressively populated the culture dishes. Following the removal of tissue explants, adherent cells exhibited a homogeneous fibroblast-like morphology (Figure 3B) and were efficiently expanded under standard culture conditions. Following exposure to 5-aza-CR for 18 h, fibroblasts displayed evident morphological changes, including a reduction in the typical elongated spindle-like shape and the acquisition of a more compact and less differentiated appearance (Figure 3C).

Gene expression analysis demonstrated molecular evidence of successful chemical reprogramming (Figure 3D). In detail, chemically reprogrammed fibroblasts (post-5-aza-CR) exhibited a significant downregulation of the fibroblast-specific markers VIM and THY1 compared to that in untreated control cells (Untreated). In parallel, 5-aza-CR-treated cells showed the onset of the pluripotency-associated transcription factors OCT4, NANOG, REX1, and SOX2, which were originally undetectable in untreated skin fibroblasts.

### 3.3 Repopulation of testicular bio-scaffolds with chemical reprogramming of human skin fibroblasts

**FIGURE 4:**
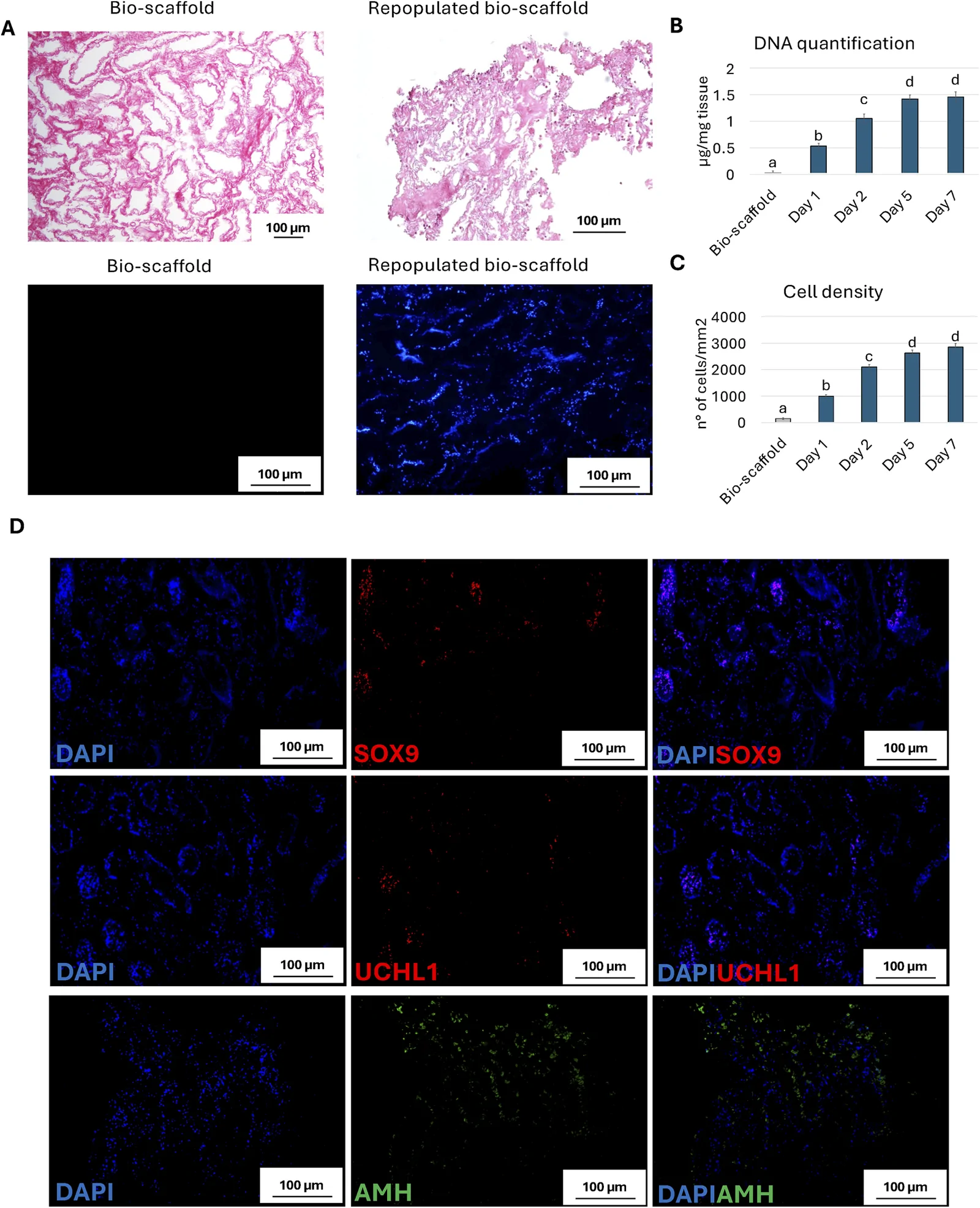
Repopulation of testicular bio-scaffolds with chemically reprogrammed human skin fibroblasts. **(A)** Representative images showing decellularized testicular bio-scaffold before and after repopulation with chemically reprogrammed human skin fibroblasts. Upper panels: H&E staining. Lower panels: DAPI staining (scale bars: 100 µm). **(B)** DNA quantification analysis showing a progressive increase in total DNA content in repopulated bio-scaffolds over the culture period. Data are expressed as the mean ± standard deviation (SD). ^a,b,c^ Different superscripts indicate significant differences (*p* < 0.05). **(C)** DAPI-based quantitative analysis confirming a statistically significant increase in cell density over time. Data are expressed as the mean ± standard deviation (SD). ^a,b,c^ Different superscripts indicate significant differences (*p* < 0.05). **(D)** Representative immunofluorescence images showing the expression of SOX9, UCHL1, and AMH in chemically reprogrammed human fibroblasts cultured onto decellularized testicular bio-scaffolds for 7 days. Nuclei were counterstained with DAPI (scale bars: 100 μm).

Chemically reprogrammed human skin fibroblasts efficiently adhered to the decellularized testicular bio-scaffolds, and subsequent culture allowed progressive colonization of the ECM (Figure 4A, upper panels). DAPI staining further demonstrated a homogeneous cellular distribution throughout the matrix (Figure 4A, lower panels). DNA quantification analysis revealed a progressive increment in the total content in repopulated bio-scaffolds during the culture period (Figure 4B). In addition, DAPI-based quantitative analysis confirmed a statistically significant increase in cell density over time (Figure 4C).

**FIGURE 5:**
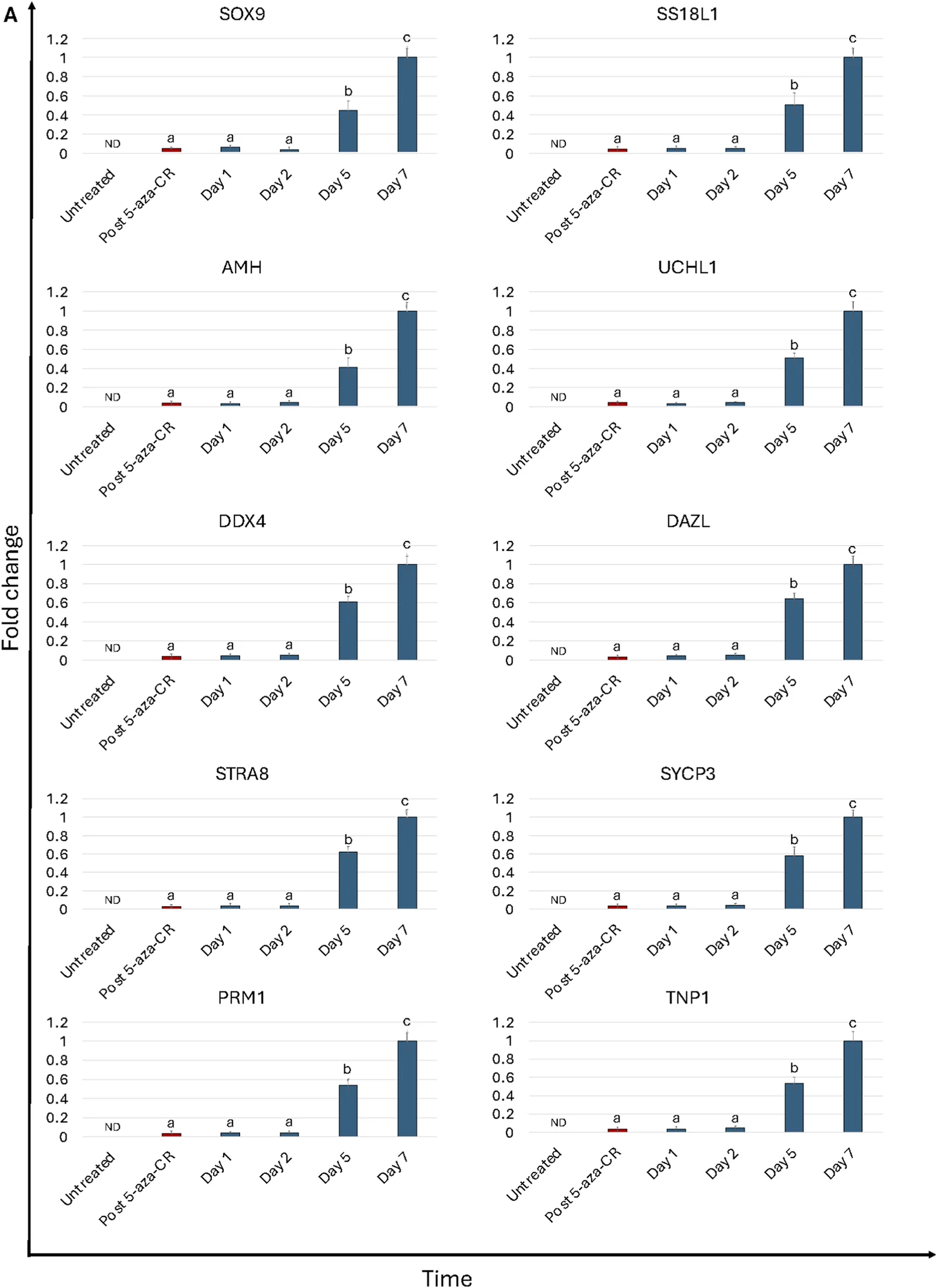
Gene expression analysis of testicular somatic and germline-associated markers (*SOX9, SS18L1, AMH, UCHL1, DDX4, DAZL, STRA8, SYCP3, PRM1, and TNP1*) in untreated skin fibroblasts (untreated), chemically reprogrammed fibroblasts (post-5-aza-CR), and at days 1, 2, 5, and 7 post-seeding onto testicular bio-scaffolds. Gene expression is presented relative to the highest expression value set to 1. ^a,b,c^Different superscripts indicate significant differences (*p* < 0.05).

Gene expression analysis demonstrated the expression of VIM and THY1 throughout the 7-day culture period (Figure 3D). However, both fibroblast-specific markers stabilized at lower expression levels compared to that in untreated cells (Figure 5). This was accompanied by the induction of genes associated with testicular somatic and progenitor-like lineages, namely, SOX9, SS18L1, AMH, UCHL1, DDX4, DAZL, STRA8, SYCP3, PRM1, and TNP1, which were originally undetectable in untreated fibroblasts (Figure 5).

Immunofluorescence analyses further confirmed the expression of testicular-associated proteins within the recellularized bio-scaffolds. In particular, cells positive for SOX9, UCHL1, and AMH were detected after 7 days of culture (Figure 4D).

## 4 Discussion

In the present manuscript, we describe the generation of testicular ECM-based bio-scaffolds capable of replicating the *in vivo* topography and the bio-mechanical and bio-chemical stimuli of the native tissue, and we demonstrate their ability to support the survival, growth, and differentiation of engrafted cells.

Testicular bio-scaffolds were generated using a three-step decellularization protocol developed in our laboratory, consisting of a freeze–thaw cycle followed by sequential incubations with SDS and Triton X-100 (Di Filippo et al., 2025). As previously demonstrated and now confirmed in the present study, this protocol effectively removes cells, cellular debris, and nuclear material from testicular tissue, while preserving both the macro- and micro-architecture of the native organ, thus maintaining tissue morphology and homogeneity without evident deformation following decellularization. Furthermore, similarly to observations reported in previous studies applying decellularization protocols to a wide range of tissues, including the heart (Rajabi-Zeleti et al., 2014), lung (Lecht et al., 2014), liver (Ghiringhelli et al., 2017; Lee et al., 2017), kidney (Yu et al., 2014), muscle (Aulino et al., 2015), trachea (Baiguera et al., 2014; Pennarossa et al., 2021b; 2023b), esophagus (Sjöqvist et al., 2014), urinary tissue (Singh et al., 2018), arteries (Kajbafzadeh et al., 2017), derma (Gilpin and Yang, 2017), intestine (Arcuri et al., 2024; Pennarossa et al., 2026), ovary (Laronda et al., 2015; Laronda, 2020; Pennarossa et al., 2020a; 2021c), and vagina (Zhang et al., 2017), in the experiments described here, the testes exhibited a marked change in color from red to white at the end of the decellularization protocol. This indicates that the adopted approach caused an effective depletion of cellular material when applied to testicular tissue as well. These findings were further supported by histological analysis, which revealed a marked reduction in basophilic staining, and by DAPI staining, which demonstrated a substantial decrease in nuclear material. Consistently, the generated testicular bio-scaffolds exhibited a residual DNA content of 0.026 ± 0.01 μg/mg tissue (equivalent to 26 ± 10 ng DNA/mg tissue). This value is well below the threshold commonly accepted for effective decellularization, which is generally considered to be 50 ng DNA/mg tissue (Crapo et al., 2011; Keane et al., 2015; Gilpin and Yang, 2017). Together with the absence of detectable nuclei in DAPI and histological analyses, these findings confirm the high efficiency of the decellularization protocol utilized and indicate that the generated bio-scaffolds meet commonly accepted criteria for effective decellularization. These data are in agreement with previous studies reporting SDS ability to successfully eliminate the cellular compartment from the native tissues when the concentration and time of exposure were optimized in an appropriate tissue-specific manner (Scarrit, 2015; Gilpin and Yang, 2017; Singh et al., 2023). Taken together, these results demonstrate the effectiveness of the decellularization protocol used in the present manuscript and indicate that the correct combination of the freeze–thaw treatment and detergent-based decellularization enables the generation of testicular bio-scaffolds with minimal residual DNA contents. However, it is important to consider that a critical aspect of the decellularization process is achieving a balance between the effective removal of cellular components and the preservation of the native ECM microarchitecture, including structural fibers and macromolecular components. In this context, the use of SDS remains controversial and needs to be further clarified. Indeed, several studies reported a detrimental effect of this detergent, with damages to structural proteins, such as collagen fibers in porcine urinary bladder (Faulk et al., 2014), caprine pancreas (Singh et al., 2023), human and porcine lung (O’Neill et al., 2013), heart valve (Zhou et al., 2010), and many other tissues (Crapo et al., 2011; Keane et al., 2015). Similarly, decrease in GAG content associated with the use of SDS has been reported in different organs, including the liver, pericardium, articular cartilage, heart, and kidney (Uygun et al., 2010; Moffat et al., 2022; Kasturi and Vasanthan, 2023). On the other hand, it is noteworthy that all the histochemical analyses performed in the present study demonstrated the preservation of collagen, elastic fibers, and glycosaminoglycans following decellularization and indicated that careful optimization of the SDS concentration and exposure time is essential to ensure efficient cell removal while preserving ECM integrity in a species- and tissue-specific manner.

These observations were further supported by SEM, which demonstrated preservation of the 3D ultrastructure of the decellularized testicular bio-scaffolds. In particular, this analysis revealed maintenance of the seminiferous tubule architecture together with an interconnected extracellular matrix network characterized by well-organized collagen fibers. Preservation of these ultrastructural features is particularly relevant, as the native spatial organization of the ECM plays a crucial role in providing biomechanical support and tissue-specific instructive cues that regulate cell adhesion, migration, and differentiation during scaffold repopulation.

Similarly, remnant detergent within the decellularized bio-scaffolds is a crucial point and may impair the subsequent recellularization and biocompatibility, both *in vitro* and *in vivo* (Morris et al., 2017). Cytotoxicity, evaluated by MTT assay, revealed no significant differences in OD values between cells co-cultured with decellularized testes and those of the CTR group. Furthermore, quantification of residual SDS content demonstrated efficient detergent removal following decellularization. These findings, together with the absence of detectable cytotoxic effects in MTT assays, indicate that residual SDS is unlikely to adversely affect scaffold biocompatibility, cell survival, or subsequent recellularization. These results are very encouraging and allow us to consider any toxic effect exerted by the bio-scaffold in culture as very unlikely. These findings further support the biocompatibility of the generated scaffold, indicating that it provides a favorable microenvironment able to sustain cell survival, growth, and differentiation. Indeed, its re-seeding with human fibroblasts demonstrated rapid cell adhesion and migration within the first 24 h, with the repopulating cells increasing in number and aggregating in cluster-like structures. Overall, these findings support and extend previous reports on decellularization strategies applied to different organs and suggest the feasibility of applying this approach to testicular tissue, leading to the generation of ECM-based scaffolds suitable for tissue engineering applications.

In this perspective, decellularized testicular bio-scaffolds were further evaluated in repopulation experiments. Specifically, we hypothesized that ECM-based scaffolds might act as a tissue-specific niche that provide differentiation cues and influence transcriptional regulatory mechanisms, thereby offering a 3D microenvironment able to direct the repopulating cell fate. To this end, human fibroblasts were chemically reprogrammed through a brief exposure to the well-known DNA demethylating agent 5-aza-CR and used for the recellularization of the testicular bio-scaffolds. In particular, a high-plasticity state was induced in terminally differentiated skin fibroblasts isolated from human healthy individuals using an 18-h exposure to 5-aza-CR, which has been previously demonstrated to reactivate pluripotency-related genes (Taylor and Jones, 1979; Jones, 1985b; Jones, 1985a; Glover et al., 1986), to induce a global DNA hypomethylation (Palii et al., 2008; Pennarossa et al., 2013; Pennarossa et al., 2014; Pennarossa et al., 2018; Pennarossa et al., 2019; Pennarossa et al., 2020b; Brevini et al., 2014; Brevini et al., 2016a; Brevini et al., 2016b; Mirakhori et al., 2015a; Mirakhori et al., 2015b; Tan et al., 2015; Chandrakanthan et al., 2016; Manzoni et al., 2016; Diomede et al., 2018) and to modulate 10–11 translocation (TET) gene transcription (Manzoni et al., 2016). In agreement with what was previously described, reprogrammed fibroblasts lost their typical elongated morphology and acquired the features distinctive of a high-plasticity phenotype, resembling those previously observed in embryonic stem cells (ESCs) (Niwa, 2007; Lai et al., 2015) and induced pluripotent stem cells (iPSCs) (Courtot et al., 2014), including a round or oval shape, enlarged nuclei, and granular, vacuolated cytoplasm. As expected, this morphological reprogrammed phenotype was also accompanied by the expression of the main pluripotency-related gene transcription, namely, OCT4, NANOG, REX1, and SOX2, which were undetectable in untreated fibroblasts, together with statistically significant downregulation of the fibroblast-related markers VIM and THY1. These data further support previously published reports that described 5-aza-CR’s ability to induce a pluripotent-like phenotype hypomethylation (Palii et al., 2008; Pennarossa et al., 2013; Pennarossa et al., 2014; Pennarossa et al., 2018; Pennarossa et al., 2019; Pennarossa et al., 2020b; Brevini et al., 2014; Brevini et al., 2016a; Brevini et al., 2016b; Mirakhori et al., 2015a; Mirakhori et al., 2015b; Tan et al., 2015; Chandrakanthan et al., 2016; Manzoni et al., 2016; Diomede et al., 2018).

Following seeding onto testicular bio-scaffolds, the cells adhered, progressively colonizing the matrix within 24 h. Bio-scaffold cell-homing ability was confirmed by H&E and DAPI staining, along with quantitative cell-density analysis, which showed a progressive increase in cell number up to day 7, when culture was arrested. These observations indicate that decellularized bio-scaffolds support the survival of engrafted cells *in vitro* over extended culture periods, as further confirmed by DNA quantification analyses showing a progressive increase in DNA content over time. Notably, marked changes in cell transcriptional profiles were observed. Gene expression analysis indicated a downregulation of the pluripotency-related genes OCT4, NANOG, REX1, and SOX2 and concomitant induction of the key testicular and germ cell-associated markers, namely, SOX9, SS18L1, AMH, UCHL1, DDX4, DAZL, STRA8, SYCP3, PRM1, and TNP1, which were undetectable in untreated fibroblasts. Notably, the transcriptional findings were further validated by immunofluorescence analyses, which confirmed the presence of SOX9-, UCHL1-, and AMH-positive cells within the recellularized bio-scaffolds. The detection of SOX9 and AMH proteins supports commitment toward testicular somatic lineages, while UCHL1 expression is consistent with the activation of germ-cell-associated molecular programs. Overall, these results support the gene expression data and further point to the ability of the testicular ECM to promote the acquisition of testicular-associated phenotypes by chemically reprogrammed fibroblasts. This indicates the activation of transcriptional programs associated with testicular cell identity. In particular, temporal analysis revealed dynamic modulation of these genes over the culture period, indicating that the testicular bio-scaffold provides instructive cues that influence the cell behavior and promote the expression of markers associated with testicular lineage commitment. Therefore, beyond supporting the survival and viability of chemically reprogrammed cells, the generated bio-scaffolds promote changes consistent with differentiation toward a testicular-associated phenotype, accompanied by a molecular shift in response to tissue-specific microenvironmental cues. It is also important to note that the decellularized bio-scaffolds obtained from the testicular tissue retain extracellular cues associated with multiple cellular niches. Consequently, chemically reprogrammed fibroblasts used to repopulate the bio-scaffolds may receive different instructive signals and undergo commitment toward distinct testicular phenotypes. In this context, the simultaneous detection of markers associated with Sertoli and germ-cell lineages is consistent with the generation of heterogeneous cell subpopulations that collectively reflect the complexity of the native testicular microenvironment. Altogether, these findings support and further expand our previous work demonstrating the ability of ovarian decellularized bio-scaffolds to provide an optimal 3D microenvironment for *ex vivo* culture of ovarian cells, supporting the maintenance of their original expression pattern. Interestingly enough, and in agreement to what is described in the present manuscript, the decellularized bio-scaffold had the ability to properly drive chemically reprogrammed cell differentiation into mature and functional granulosa cells (Pennarossa et al., 2021a).

Overall, the findings presented in this study demonstrate that decellularized testicular bio-scaffolds provide an optimal 3D microenvironment for *ex vivo* cell culture. In particular, these scaffolds support the survival and viability of chemically reprogrammed cells and promote transcriptional changes consistent with differentiation toward a testicular-associated phenotype, promoting a transcriptional shift in response to tissue-specific microenvironmental cues. Although these changes are encouraging, the 7-day culture period utilized in the present study does not allow definitive conclusions regarding the long-term maintenance of the induced phenotype. Therefore, further studies will be required to determine whether the observed expression of testicular-associated markers is maintained over time and reflects stable commitment toward testicular cell phenotypes. In addition, functional analyses will be necessary to confirm the biological properties and functionality of the resulting cell populations.

In conclusion, the bioengineering strategy proposed here paves the way for the development of physiologically relevant 3D platforms for analyzing male infertility, restoring endocrine function, and supporting toxicological and drug screening applications, along with future regenerative and transplantation studies.

## Data Availability

The original contributions presented in the study are included in the article/supplementary material; further inquiries can be directed to the corresponding author.
